# Enhanced expression of PD-1 and other activation markers by CD4+ T cells of young but not old patients with metastatic melanoma

**DOI:** 10.1007/s00262-018-2148-6

**Published:** 2018-03-15

**Authors:** Rob R. H. van den Brom, Kornelis S. M. van der Geest, Elisabeth Brouwer, Geke A. P. Hospers, Annemieke M. H. Boots

**Affiliations:** 1 Department of Medical Oncology, University Medical Center Groningen, University of Groningen, Groningen, The Netherlands; 2 Department of Rheumatology and Clinical Immunology, University Medical Center Groningen, University of Groningen, Hanzeplein 1, 9700 RB Groningen, The Netherlands

**Keywords:** Melanoma, Immunosenescence, Aging, PD-1, CTLA-4

## Abstract

The biological behavior of melanoma is unfavorable in the elderly when compared to young subjects. We hypothesized that differences in T-cell responses might underlie the distinct behavior of melanoma in young and old melanoma patients. Therefore, we investigated the circulating T-cell compartment of 34 patients with metastatic melanoma and 42 controls, which were classified as either young or old. Absolute numbers of CD4+ T cells were decreased in young and old melanoma patients when compared to the age-matched control groups. Percentages of naive and memory CD4+ T cells were not different when comparing old melanoma patients to age-matched controls. Percentages of memory CD4+ T cells tended to be increased in young melanoma patients compared to young controls. Proportions of naive CD4+ T cells were lower in young patients than in age-matched controls, and actually comparable to those in old patients and controls. This was accompanied with increased percentages of memory CD4+ T cells expressing HLA-DR, Ki-67, and PD-1 in young melanoma patients in comparison to the age-matched controls, but not in old patients. Proportions of CD45RA−FOXP3^high^ memory regulatory T cells were increased in young and old melanoma patients when compared to their age-matched controls, whereas those of CD45RA+FOXP3^low^ naive regulatory T cells were similar. We observed no clear modulation of the circulating CD8+ T-cell repertoire in melanoma patients. In conclusion, we show that CD4+ T cells of young melanoma patients show signs of activation, whereas these signs are less clear in CD4+ T cells of old patients.

**Electronic supplementary material:**

The online version of this article (10.1007/s00262-018-2148-6) contains supplementary material, which is available to authorized users.

## Introduction

Melanoma is an aggressive form of skin cancer with frequent metastases towards other organs. The incidence of melanoma in Europe is currently on the rise [1]. Among adolescents and young adults, melanoma is the most prevalent type of cancer in women and ranks second in men [2]. Nevertheless, melanoma is largely a disease of the elderly, as 43% of all newly diagnosed patients are 65 years or older. In addition, the median age at diagnosis is 64 years for males and 57 for females [3]. Importantly, the biological behavior of melanoma differs between young and old patients. Old patients more frequently present with unfavorable prognostic tumor factors as evidenced by a higher Breslow’s thickness, a higher occurrence of histological ulcerative tumors, and a higher mitotic activity [4–6]; and, finally, a worse disease-specific survival [6]. Currently, it is unclear why the biological behavior of melanoma differs in young and old patients.

Ample evidence indicates that the immune system plays a key role in the outcome of melanoma. Spontaneous regression occurs in 3.7*–*15% of primary melanomas. Even for metastatic melanoma, one in every 400 patients reaches a spontaneous complete remission [7]. Immune checkpoint inhibitors like the anti-cytotoxic T-lymphocyte-associated protein 4 (CTLA-4) antibody ipilimumab and the anti-programmed cell death protein 1 (PD-1) antibodies nivolumab and pembrolizumab have demonstrated remarkable efficacy in boosting T-cell responses against metastatic melanoma [8]. For the combination of ipilimumab and nivolumab, a response rate as high as nearly 60% was reported in 2015 [9]. Currently, no validated biomarkers are commonly used to select melanoma patients for treatment with checkpoint inhibitors.

Aging of the immune system might be a factor contributing to the unfavorable behavior of melanoma in the elderly. Both the innate and adaptive immune arms of the immune system are affected by aging [10, 11]. These changes have been linked to the increased susceptibility for infections and various types of cancer in the elderly [12–14]. T-cell responses might be compromised in the elderly due to various perturbations, such as reduced numbers and diversity of naïve T cells, skewing of the memory T-cell receptor repertoire, poor cytokine secretion, and functional exhaustion of the memory compartment [10, 11, 15–18]. Moreover, numbers of regulatory T cells increase with aging. Regulatory T cells inhibit immune responses and are essential for preventing autoimmunity. In the context of cancer, however, these cells may dampen anti-tumor responses. It might, therefore, be possible that aging of cellular immunity underlies the unfavorable behavior of melanoma in the elderly.

In the current study, we, therefore, investigated the circulating T-cell compartments of young and old melanoma patients. For comparison, we recruited a cohort of aged-matched healthy controls. A comprehensive analysis of activation, proliferation, and differentiation markers, checkpoint molecules, and regulatory T-cell transcription factors shows that CD4+ T cells of young melanoma patients show signs of an ongoing immune response, whereas these signs are lacking in CD4+ T cells of old melanoma patients.

## Materials and methods

### Study subjects

Peripheral blood was obtained from 34 systemic treatment-naive, metastatic melanoma patients, who were either classified as ‘young’ when ≤ 50 years of age (*n* = 11) or ‘old’ when ≥ 65 years of age (*n* = 18). For three patients, only lymphocyte true count could be performed due to logistic reasons. In addition, blood samples were obtained from 42 age-matched healthy controls that were young (*n* = 13) or old (*n* = 39). Health of the control subjects was assessed by health assessment questionnaires, physical examination, and blood tests as previously described [11]. Melanoma patients using immune-modulating drugs or having infections, other types of malignant disease, or autoimmune disease were excluded from the study.

### Flow cytometry

Peripheral blood mononuclear cells (PBMC) were isolated by density centrifugation with Lymphoprep (Axis-Shield). PBMC or whole blood samples were stained with the following fluorochrome-conjugated monoclonal antibodies: CD3-efluor605, CD4-efluor450, CD27-APC-efluor780, HLA-DR-efluor780, CD45RA-efluor605, FOXP3-PE (eBioscience), CD4-APC-H7, CD8-Percp, CD8-PE-Cy7, CD31-AF647, CD45RO-FITC, CD45RO-PE-Cy7, CCR7-PE-Cy7, Ki-67-Percp-cy5.5, CTLA-4-BV421 (BD Biosciences), PD-1-PE, CD28-AF700 (Biolegend), and CD161-PE (Miltenyi Biotec). Intracellular staining for FOXP3, Helios, Ki-67, and CTLA-4 was performed after cells were permeabilized with a FOXP3 staining buffer set according to instructions of the manufacturer (eBioscience). Whole blood samples were treated with BD lysing solution according to the instructions of the manufacturer (BD Biosciences). Stained samples were analyzed on a LSR-II flow cytometer (BD Biosciences). Analysis was performed with Kaluza Flow Analysis Software (Beckman Coulter). Absolute numbers of CD3+ T cells, CD4+ T cells, CD8+ T cells, B cells, and NK cells were determined according to the MultiTest TruCount method (BD Biosciences), as described by the manufacturer. TruCount samples were measured on a FACSCanto-II (BD Biosciences) and analyzed with FACSCanto Clinical Software (BD Biosciences).

### Statistics

Demographics and baseline characteristics of all patients were summarized using descriptive statistics. The Mann Whitney *U* Test was used to compare different groups. Analyses were performed with GraphPad Prism 5.0. Two-tailed *p* values < 0.05 were considered significant.

## Results

### Subjects characteristics and lymphocyte numbers

Baseline characteristics of the melanoma patients and healthy controls are shown in supplemental Table 1 and supplemental Table 2, respectively. The time between development of metastases after discovery of the primary tumor was shorter in old compared to young melanoma patients, albeit not statistically significant. Markers of systemic inflammation—erythrocyte sedimentation rate and C-reactive protein—tended to be higher in young patients than in old patients. Absolute numbers of CD3+ T cells were lower in melanoma patients when compared to their aged-matched healthy controls (Table 1). This difference could be explained by a numerical decline of CD4+ T cells in melanoma patients, whereas numbers of circulating CD8+ T cells were similar in patients and controls. Absolute numbers of B cells were decreased in young and old melanoma patients compared to the aged-matched control. Numbers of NK cells were similar in patients and controls. Thus, absolute numbers of circulating CD4+ T cells and B cells are altered in patients with metastatic melanoma.

**Table 1:** True counts of peripheral lymphocyte subsets shown for young and old metastatic melanoma patients compared to age-matched healthy controls

	Young HC (*n* = 13)	Young melanoma patient (*n* = 13)	Old HC (*n* = 28)	Old melanoma patients (*n* = 18)
CD3+ count, × 10^9^/L	1.12 (0.78*–*1.59)	0.89 (0.47*–*1.59)*	1.28 (0.55*–*2.34)	0.93 (0.50*–*2.28)*
CD4+ count, × 10^9^/L	0.79 (0.18*–*0.99)	0.51 (0.24*–*0.95)^a^	0.89 (0.33*–*1.43)	0.53 (0.31*–*1.21)**
CD8+ count, × 10^9^/L	0.32 (0.19*–*0.74)	0.30 (0.10*–*0.57)	0.34 (0.10*–*1.25)	0.24 (0.06*–*1.00)
B cell counts,× 10^9^/L	0.19 (0.08*–*0.50)	0.12 (0.03*–*0.22)*	0.18 (0.06*–*0.50)	0.13 (0.04*–*4.19)^b^
NK cell counts, × 10^9^/L	0.15 (0.06*–*0.44)	0.19 (0.06*–*0.37)	0.31 (0.07*–*0.65)	0.21 (0.03*–*0.51)

*HC* healthy controls, *NK* natural killer

*p < 0.05 or **p < 0.01

^a^*p* value: 0.057

^b^*p* value: 0.051

For melanoma patients, subsequent treatment after inclusion and survival outcomes are provided in supplemental Table 3.

### T-cell differentiation subsets

**Fig. 1:**
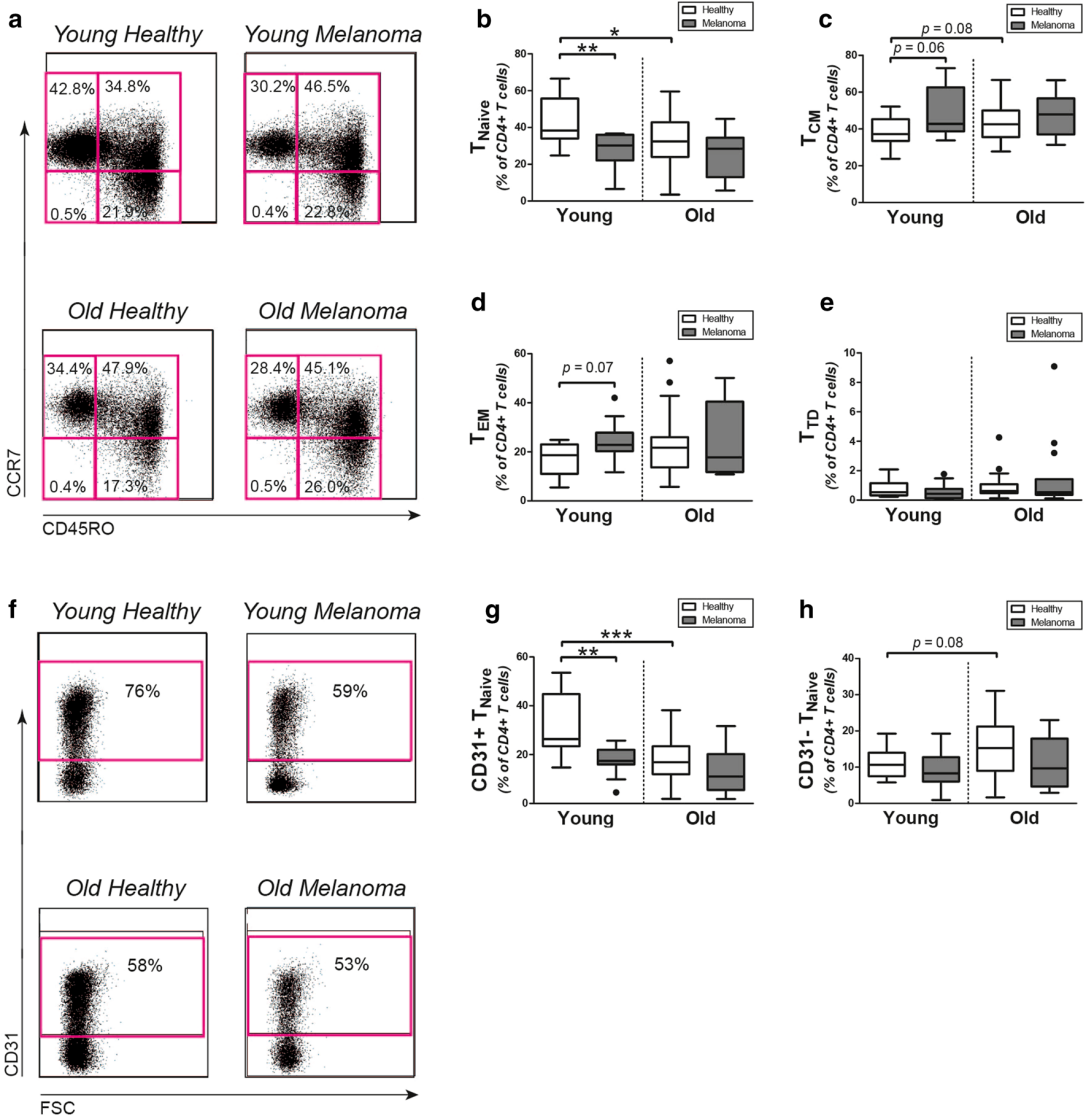
CD4+ T-cell differentiation subsets in melanoma patients and controls. **a** Representative flow cytometric staining of CD45RO and CCR7 in CD4+ T cells in melanoma patients and age-matched controls. Percentages of **b** CD45RO−CCR7+CD4+ T_Naive_ cells, **c** CD45RO+CCR7+CD4+ T_CM_ cells, **d** CD45RO+CCR7−CD4+ T_EM_, and **e** CD45RO−CCR7−CD4+ T_TD_ cells in young controls (*n* = 13), young patients (*n* = 11), old controls (*n* = 39), and old patients (*n* = 15). **f** Representative flow cytometric staining for CD31 in CD4+ T cells in melanoma patients and healthy controls. Percentages of **g** CD31+ thymic emigrant CD4+ T_Naive_ cells and **h** CD31− central CD4+ T_Naive_ cells in the same patients and controls. Statistical significance is indicated as *p < 0.05, **p < 0.01 and ***p < 0.001

We investigated if the lower number of CD4+ T cells in melanoma patients resulted from a decline of particular T-cell differentiation subsets. Therefore, we further divided the CD4+ T cells compartment into CD45RO−CCR7+ naive (T_Naive_), CD45RO+CCR7+ central memory (T_CM_), CD45RO+CCR7− effector memory (T_EM_), and CD45RO−CCR7− terminally differentiated (T_TD_) cells (Fig. 1a). Proportions of CD4+ T_Naive_ cells were decreased in young melanoma patients when compared to age-matched healthy controls (Fig. 1b). Proportions of CD4+ T_Naive_ cells in young melanoma patients were actually similar to those in old patients and controls. We observed trends for increased proportions of CD4+ T_CM_ and T_EM_ cells in young melanoma patients versus age-matched controls (Fig. 1c, d), whereas proportions of CD4 T_TD_ cells were similar in young patients and controls (Fig. 1e). The percentages of all CD4+ T-cell differentiation subsets were similar in old melanoma patients and age-matched controls. We obtained similar results when CD4+ T_Naive_ and CD4+ T_TD_ cells were more stringently defined as CD45RO−CCR7+CD27+CD28+ and CD45RO−CCR7−CD27−CD28− cells, respectively (Supplemental Fig. 1). Although percentages of CD8+ T_Naive_ cells tended to be somewhat lower in young melanoma patients versus young healthy controls, we observed no clear differences between CD8+ T-cell differentiation subsets of melanoma patients and healthy controls (Supplemental Figs. 2a, 2b, 2c and 2d).

As CD4+ T_Naive_ cells were found reduced in young melanoma patients, we next determined if CD31+ thymic emigrant CD4+ T_Naive_ cells or post-thymically expanded CD31− central CD4+ T_Naive_ cells were decreased in young melanoma patients (Fig. 1f). Proportions of CD31+ thymic emigrant CD4+ T_Naive_ cells were decreased in young patients when compared to age-matched controls (Fig. 1g). Young melanoma patients were actually demonstrating similar low proportions of these cells, as old patients and controls. In contrast, proportions of post-thymically expanded CD31– central CD4+ T_Naive_ cells were comparable in young and old melanoma patients versus the age-matched controls (Fig. 1h). Thus, the CD4+ T_Naive_ cell compartment of young melanoma patients resembled those of old patients and controls, rather than that of young healthy controls.

### Expression of activation and proliferation markers by circulating CD4+ T cells

**Fig. 2:**
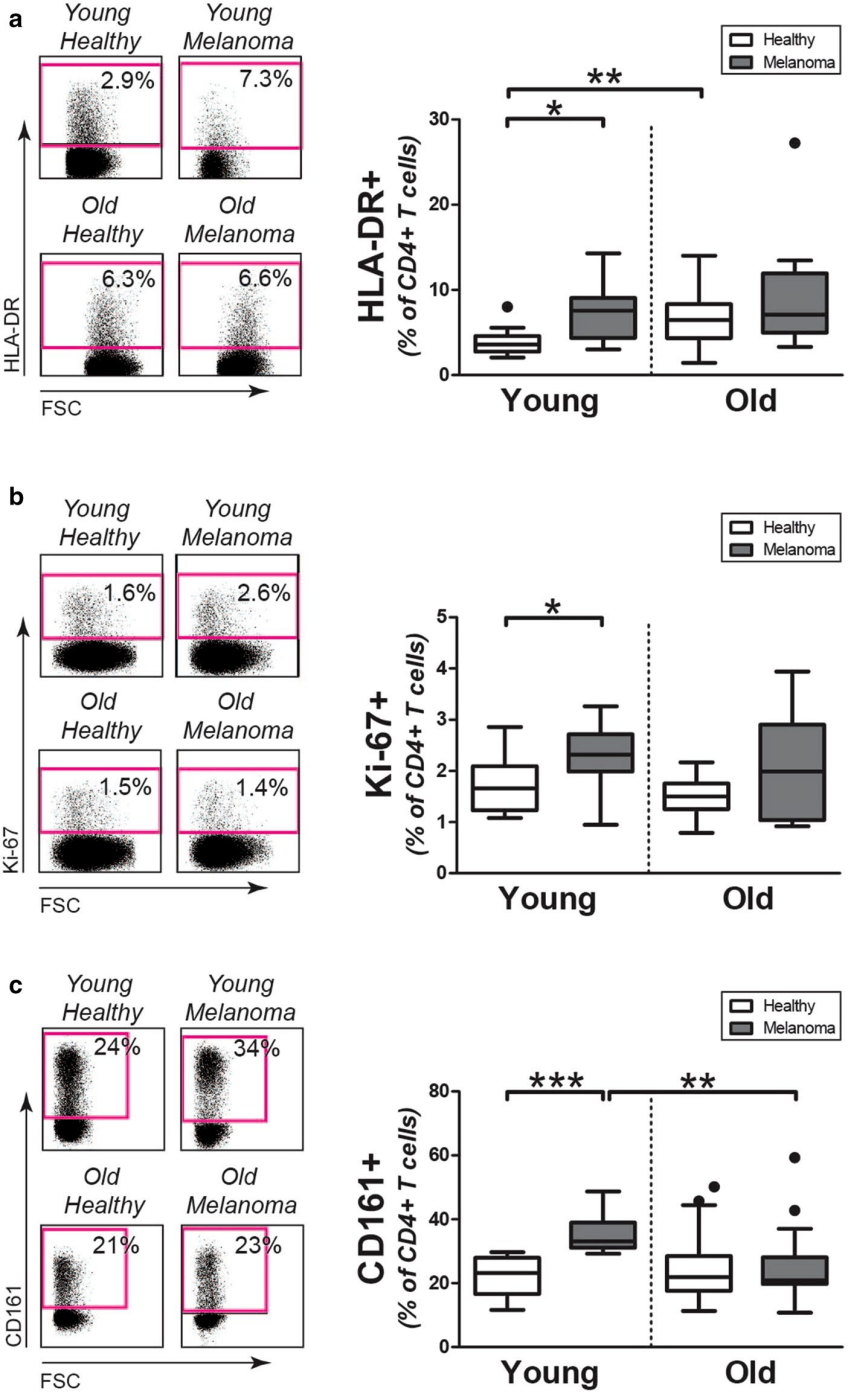
Activation and proliferation of CD4+ T cells in melanoma patients and controls. **a** Left panel: representative staining of HLA-DR on CD4+ T cells of patients and controls. Right panel: percentages of HLA-DR4+CD4+ T cells in young controls (*n* = 12), young patients (*n* = 10), old controls (*n* = 34), and old patients (*n* = 15). **b** Left panel: representative staining of intracellular Ki-67 in CD4+ T cells of patients and controls. Right panel: percentages of Ki-67+CD4+ T cells in young controls (*n* = 10), young patients (*n* = 10), old controls (*n* = 10), and old patients (*n* = 10). **c** Left panel: representative staining of CD161 on CD4+ T cells of patients and controls. Right panel: percentages of CD161+CD4+ T cells in young controls (*n* = 13), young patients (*n* = 11), old controls (*n* = 39), and old patients (*n* = 15). Statistical significance is indicated as *p < 0.05, **p < 0.01, and ***p < 0.001

We studied the activation status of CD4+ T cells in the young and old melanoma patients by determining the percentage of HLA-DR expressing cells (Fig. 2a). Percentages of HLA-DR expressing cells were increased among CD4+ T cells of young melanoma patients when compared to those in young controls. Proportions of HLA-DR expressing CD4+ T cells in young melanoma patients resembled those in old patients and controls.

In addition, we determined the percentage of proliferating CD4+ T cells by analyzing these cells for expression of Ki-67 (Fig. 2b). The percentage of Ki-67 expressing cells was higher in CD4+ T cells of young melanoma patients than those of age-matched healthy controls. In contrast, no modulation of Ki-67 was observed in CD4+ T cells of old melanoma patients when compared to their age-matched controls. No substantial increase of Ki-67 expression was observed among CD8+ T cells of melanoma patients, although the percentage of Ki-67 expressing cells tended to be slightly increased among CD8+ T cells of old melanoma patients versus old healthy controls (Supplemental Fig. 2e).

We also assessed CD4+ T cells for expression of CD161 (Fig. 2c), a killer cell lectin-like receptor that identifies a population of highly pro-inflammatory cells capable of producing interferon-γ and tumor necrosis factor-α [19]. Young melanoma patients showed an increase of CD161 expressing CD4+ T cells compared to young controls. In contrast, the percentage of CD161 expressing cells was similar in old melanoma patients and age-matched controls. In addition, percentages of CD161 expressing CD8+ T cells were similar in melanoma patients, irrespective of age (Supplemental Fig. 2f). Thus, circulating CD4+ T cells of young melanoma patients show clear signs of an ongoing immune response, whereas these signs are lacking in CD4+ T cells of old melanoma patients.

### PD-1 and CTLA-4 expression by CD4+ T cells

**Fig. 3:**
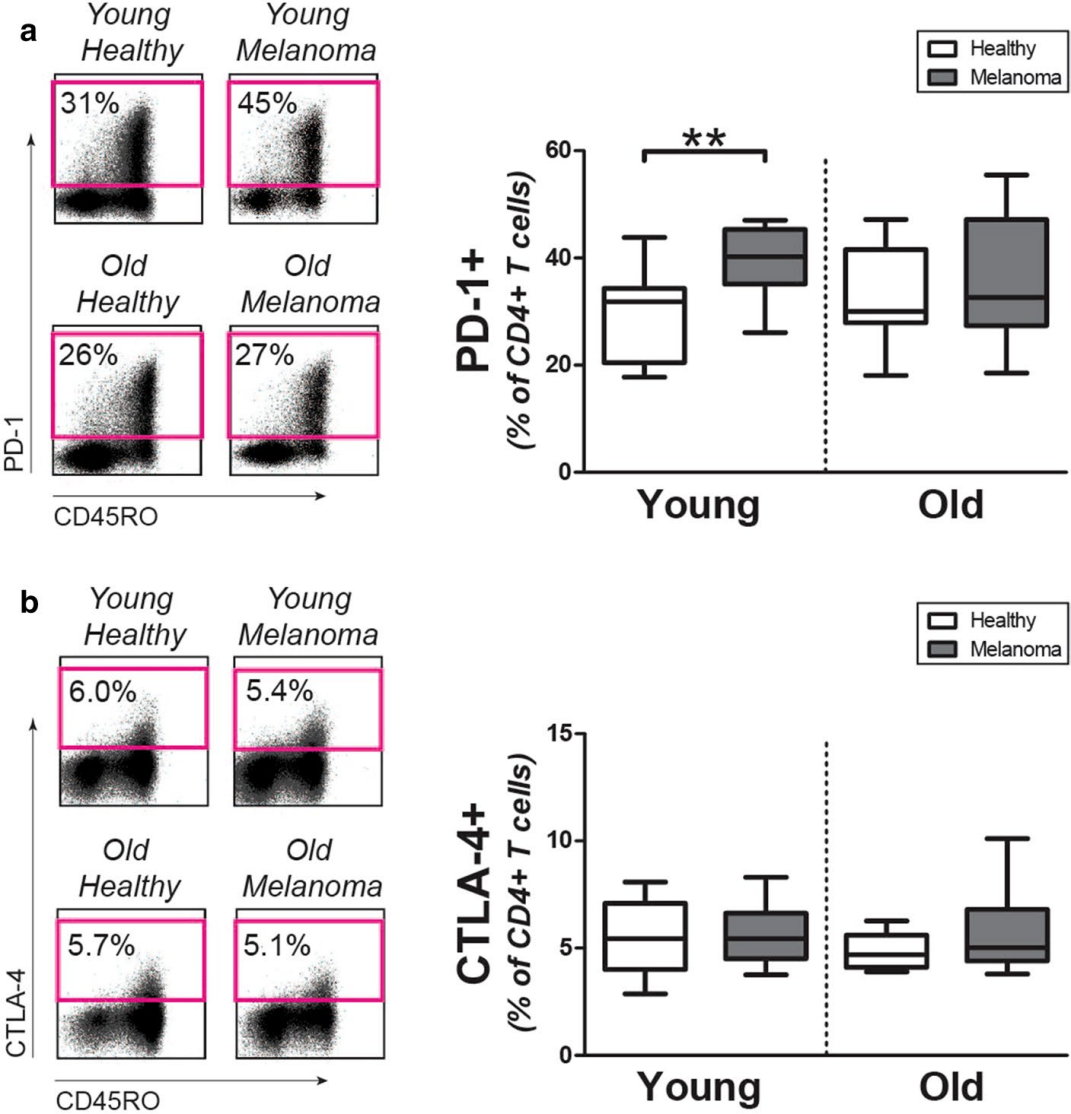
Expression of checkpoint molecules by CD4+ T cells of melanoma patients and controls. **a** Left panel: representative staining for PD-1 on CD4+ T cells of patients and controls. Right panel: percentages of PD-1+CD4+ T cells in young controls (*n* = 10), young patients (*n* = 10), old controls (*n* = 10), and old patients (*n* = 10). **b** Left panel: representative staining of intracellular CTLA-4 in CD4+ T cells of patients and controls. Right panel: percentages of CTLA-4+CD4+ T cells in the same donors as mentioned in **a**. Statistical significance is indicated as **p < 0.01

We determined if CD4+ T cells of young and old melanoma patients show increased expression of the checkpoint molecules PD-1 and CTLA-4. Percentages of PD-1 expressing cells were increased in young melanoma patients when compared to age-matched controls (Fig. 3a). In contrast, the percentage of PD-1 expressing CD4+ T cells was not modulated in old melanoma patients. The percentage of CTLA-4 expressing cells CD4+ T cells was similar in melanoma patients and controls, both in young subjects and old subjects (Fig. 3b). Percentages of PD-1 and CTLA-4 expressing CD8+ T cells were comparable in melanoma patients and healthy controls, irrespective of age (Supplemental Figs. 2g and 2 h). Taken together, the CD4+ T-cell compartment of young melanoma patients, but not old melanoma patients, shows increased expression of the checkpoint inhibitor PD-1 but not CTLA-4.

### Regulatory T cells

**Fig. 4:**
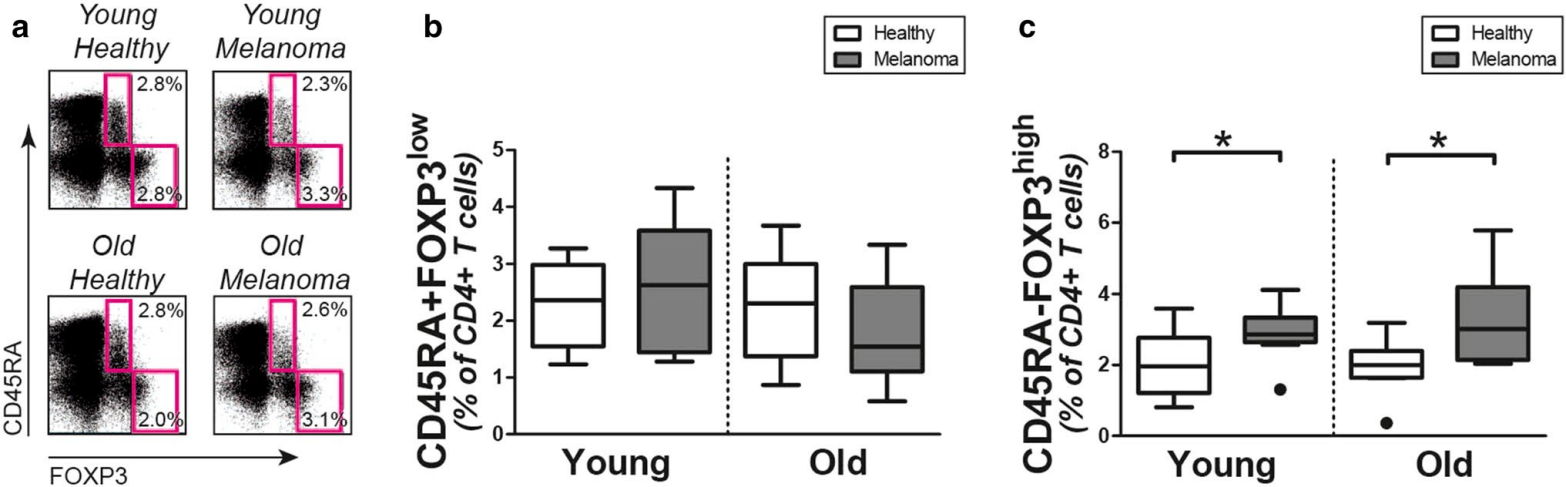
Regulatory T-cell frequencies in melanoma patients and controls. **a** Representative staining for intracellular FOXP3 and CD45RA in CD4+ T cells of patients and controls. Percentages of **b** CD45RA+FOXP3^low^ naive regulatory T cells and **c** CD45RA−FOXP3^high^ memory regulatory T cells in young controls (*n* = 10), young patients (*n* = 10), old controls (*n* = 10), and old patients (*n* = 10). Statistical significance is indicated as *p < 0.05

Finally, we questioned if numbers of regulatory T cells are modulated in melanoma patients. Therefore, we assessed the proportions of CD45RA+FOXP3^low^ naive regulatory T cells and CD45RA−FOXP3^high^ memory regulatory T cells in the peripheral CD4+ T-cell compartment of patients and controls (Fig. 4a). The proportions of CD45RA+ FOXP3^low^ naive regulatory T cells were, irrespective of age, comparable in melanoma patients and healthy controls (Fig. 4b). In contrast, we observed a clear increase of CD45RA-FOXP3^high^ memory regulatory T cells in young and old melanoma patients when compared to their age-matched controls (Fig. 4c). Thus, we observed preferential expansion of CD45RA−FOXP3^high^ memory regulatory T cells in patients with metastatic melanoma.

## Discussion

Our findings indicate a temperate CD4+ T-cell response in the peripheral blood of old melanoma patients, whereas CD4+ T cells of young melanoma patients showed prominent signs of activation, proliferation, and differentiation. The notion of an ongoing immune response in young melanoma patients is further substantiated by the decrease of thymic emigrant CD4+ T_Naive_ cells and the concomitant expansion of T_CM_ and inflammatory T_EM_ when compared to age-matched controls. Interestingly, proportions of CD4+ T_Naive_ cells in young melanoma patients were comparable to those in the old patients and controls, suggesting a melanoma-induced immune response. Thus, our findings suggest poor activation of peripheral CD4+ T cells in old melanoma patients, whereas the CD4+ T_Naive_ cell pool shows signs of premature contraction in young melanoma patients. The latter finding may be due to chronic stimulation with melanoma antigens.

The reduced activation status of circulating CD4+ T cells in old melanoma patients might contribute to the worse biological behavior and survival of melanoma in the elderly [6]. CD4+ T cells play a central role in anti-tumor responses and empower tumor-specific CD8+ T cells to gain their full cytotoxic phenotype. It remains to be elucidated why CD4+ T cells respond poorly to melanoma in the elderly. Both CD4+ T-cell inherent changes and functional impairment of antigen presenting cells are likely relevant. Remarkably, therapeutic melanoma trials with checkpoint inhibitors directed to CTLA-4 or PD-1 that prospectively stratify patients for age to assess for differences in outcome report that the response is independent of age [20–23]. One explanation for the latter finding might be the substantial selection bias in these therapeutic studies towards fit elderly with a more indolent disease course.

Although the CD4+ T cells of young melanoma patients showed clear signs of activation and proliferation, these subjects all had metastatic disease. This means that their immune system has failed to prevent disease progression and the degree of activation is, therefore, proven to be insufficient. Remarkably, we observed low proportions of CD4+ T_Naive_ cells in young melanoma patients in comparison to age-matched controls. The proportions of these cells were actually comparable to those in old patients and controls. Interestingly, this premature contraction of the CD4+ T_Naive_ cell pool in young melanoma patients could be entirely attributed to a decrease of CD31+ thymic emigrant CD4+ T_Naive_ cells. Although it is unclear if this premature contraction has developed due to or prior to disease, it likely compromises CD4+ T-cell immunity against the full spectrum of melanoma antigens.

We observed increased expression of PD-1 on circulating CD4+ T cells in young melanoma patients. This is an interesting finding, as PD-1 blocking therapy has proven successful in melanoma patients [21, 22]. PD-1 is an inhibitory receptor expressed by memory T cells and an early marker of exhausted T cells [24]. The increased expression of PD-1 on CD4+ T cells in young patients likely mirrors the activation of these cells. In contrast, we observed no clear modulation of CTLA-4 in CD4+ T cells of melanoma patients and healthy controls. Baseline signatures of peripheral blood biomarkers are studied to predict response to immune checkpoint inhibitors [25]. For example, decreasing levels of CD4+CD25+FOXP3+ regulatory T cells during ipilimumab therapy are associated with a favorable response [26]. Whether peripheral baseline PD-1 or CTLA-4 expression levels are useful to incorporate in a predictive biomarker signature is currently unclear.

Proportions of memory regulatory T cells were increased in both young and old melanoma patients. Previously, CD45RA+ FOXP3^low^ naive regulatory T cells and CD45RA−FOXP3^high^ memory regulatory T cells have been identified in the circulation of humans [27]. These two regulatory T-cell populations should be discriminated from CD45RA−FOXP3^low^ effector T cells, which lack suppressive functions. We here show that proportions of naive regulatory T cells are unaltered in melanoma patients. In contrast, proportions of memory regulatory T cells were increased in the circulation of young and old melanoma patients. It has been shown that memory regulatory T cells are immune-capable of migrating towards non-lymphoid tissues, including the skin [28]. Therefore, the expansion of memory regulatory T cells might be an unfavorable event in melanoma patients.

We are aware that our findings do not necessarily reflect immune responses at the tumor site in melanoma patients. Brisk tumor-infiltrating lymphocytes and high lymphocyte tumor distribution and density in melanoma are associated with improved disease-specific survival [29]. A study with tumor tissue samples from 147 metastatic melanoma patients showed an independent positive association between overall survival and higher counts of CD8+ T cells and PD-1 expressing cells [30]. CD4+ T-cell and regulatory T-cell counts were not predictive of survival. However, these cells may primarily fulfill their functions outside the tumor site, for instance in surrounding secondary or tertiary lymphoid structures. It would, therefore, be interesting to study CD4+ T cells in lymphoid tissues of melanoma patients.

In conclusion, our findings indicate that circulating CD4+ T cells in young patients with metastatic melanoma are quite strongly activated, whereas CD4+ T cells of old melanoma patients seem relatively dormant. This difference might contribute to unfavorable behavior of melanoma in the elderly. In addition, our findings suggest premature contraction of the CD4+ T_Naive_ cell compartment in young patients with metastatic melanoma.

### Electronic supplementary material

Below is the link to the electronic supplementary material.

Supplementary material 1 (PDF 1940 KB)

## Abbreviations

CTLA-4: Cytotoxic T-lymphocyte-associated protein 4
PBMC: Peripheral blood mononuclear cells
PD-1: Programmed cell death protein 1
T_CM_ cell: CD45RO+CCR7+CD4+ central memory T cell
T_EM_ cell: CD45RO+CCR7−CD4+ effector memory T cell
T_Naive_ cell: CD45RO−CCR7+CD4+ naive T cell
T_TD_ cell: CD45RO−CCR7−CD4+ terminally differentiated T cell
