# Supplementary material for: Enhanced expression of PD-1 and other activation markers by CD4+ T cells of young but not old patients with metastatic melanoma
Source: Cancer Immunol Immunother. 2018 Mar 15;67(6):925–33. doi: 10.1007/s00262-018-2148-6 (PMC5951899; doi:10.1007/s00262-018-2148-6)
Supplement: Supplementary file 1 — Supplementary material 1 (PDF 1940 KB) [file 262_2018_2148_MOESM1_ESM.pdf]

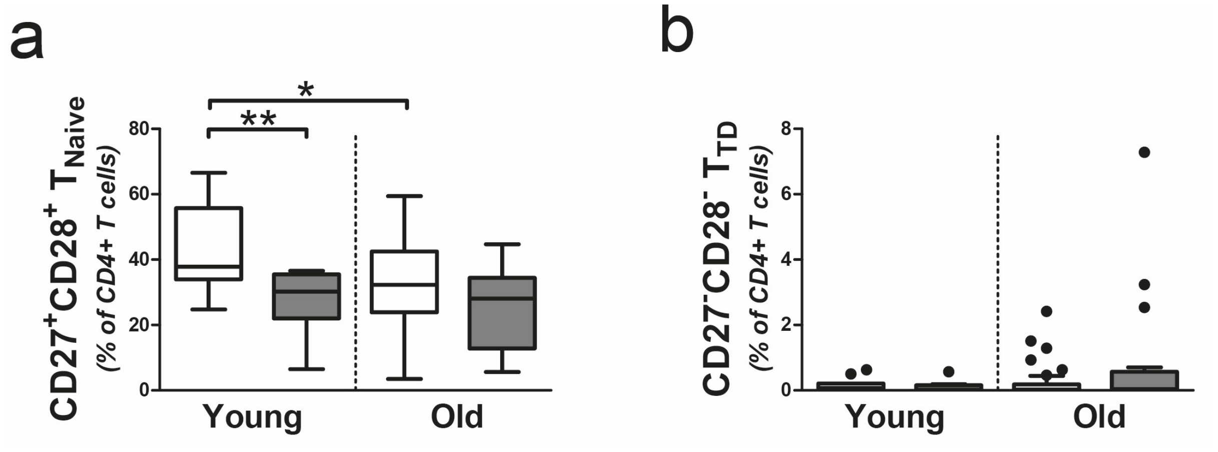

Supplemental Figure 1. Percentages of (**a**) CD27+CD28+CD45RO-CCR7+ CD4+ T_{Naive} cells and (**b**) CD27-CD28-CD45RO-CCR7- CD4+ T_{TD} cells in young controls (*n* = 13), young patients (*n* = 11), old controls (*n* = 39) and old patients (*n* = 15). Statistical significance is indicated as * $p < 0.05$ and *** $p < 0.001$.

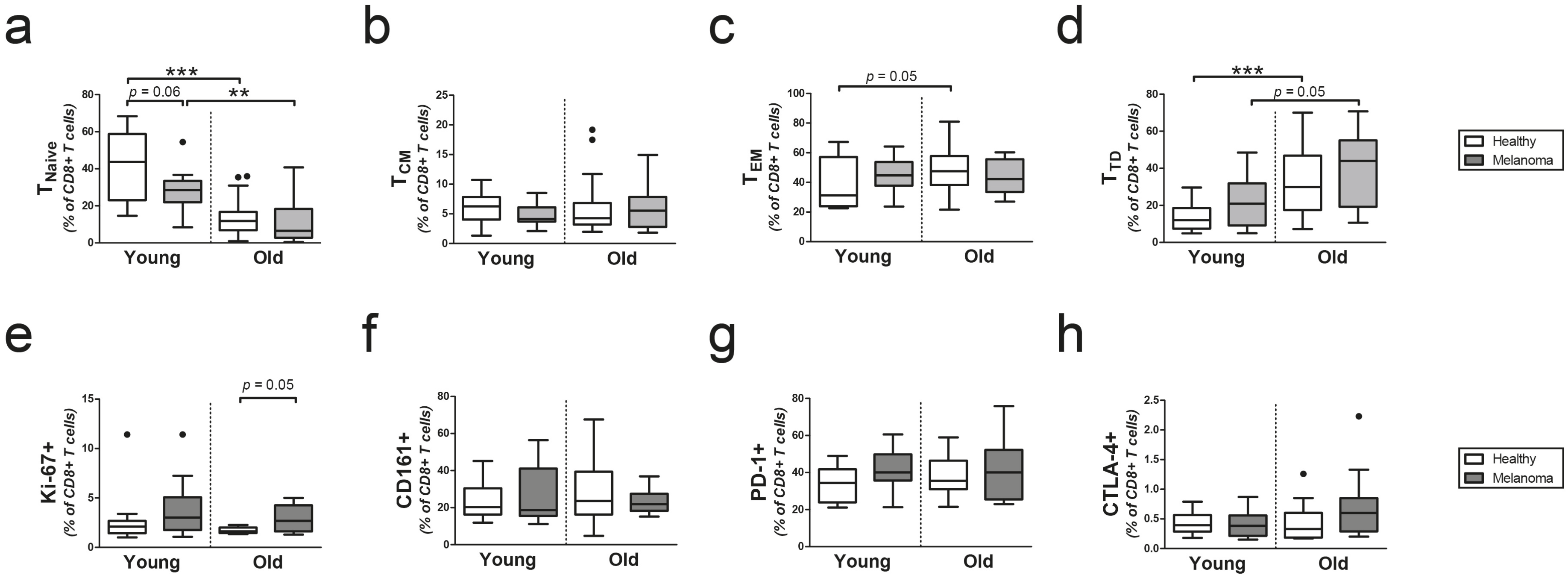

Supplemental Figure 2. Percentages of (**a**) CD45RO-CCR7+ CD8+ T_{Naive} cells, (**b**) CD45RO+CCR7+ CD8+ T_{CM} cells, (**c**) CD45RO+CCR7- CD8+ T_{EM} and (**d**) CD45RO-CCR7- CD8+ T_{TD} cells in young controls (n = 13), young patients (n = 11), old controls (n = 39) and old patients (n = 15). (**e**) Percentages of Ki-67+ CD8+ T cells in young controls (n = 10), young patients (n = 10), old controls (n = 10), old patients (n = 10). (**f**) Percentages of CD161+ CD8+ T cells in young controls (n = 13), young patients (n = 11), old controls (n = 39) and old patients (n = 15). (**g**) Percentages of PD-1+ CD8+ T cells in young controls (n = 10), young patients (n = 10), old controls (n = 10), old patients (n = 10). (**h**) Percentages of CTLA-4+ CD4+ T cells in the same donors as mentioned in (**g**). Statistical significance is indicated as * $p < 0.05$, ** $p < 0.01$ and *** $p < 0.001$.

Supplemental Table 1. Characteristics of young and old metastatic melanoma patients prior to systemic treatment.

	young melanoma patients<br>*n* = 13	old melanoma patients<br>*n* = 18
median age in years (range)	41 (19–48)	75 (65–88)
gender (m/f)	9/4	8/10
median time to metastases (months)	46	29
Breslow's thickness (mm)	1.9 ± 0.75	3.0 ± 0.48
M1a/M1b/M1c (%)	15.4/15.4/69.2	22.2/16.7/61.1
BRAF mutation (%)	77%	65%
S-100B (µg/L)	0.48 ± 0.16	1.08 ± 0.33
LDH (U/L)	201 ± 34	209 ± 18
ESR (mm/h)	32 ± 8	25 ± 5
CRP (mg/L)	29 ± 16	8 ± 2
hemoglobin (mmol/L)	8.4 ± 0.4	8.6 ± 0.2

The values shown represent a mean ± standard error of the mean unless otherwise indicated. *BRAF* = the gene encoding the serine/threonine-protein kinase B-Raf, S-100B = S100 calcium binding protein B, LDH = lactate dehydroxygenase, ESR = erythrocyte sedimentation rate, CRP = C-reactive protein.

Supplemental Table 2. Characteristics of the healthy controls.

	young healthy controls<br>*n* = 13	old healthy controls<br>*n* = 39
median age in years (range)	38 (19–53)	73 (65–92)
gender (m/f)	2/11	15/24
hemoglobin (mmol/L)	8.3 ± 0.2	10.8 ± 2.1
leukocyte count 10^9/L	4.9 ± 0.4	6.2 ± 0.2
thrombocyte count 10^9/L	232 ± 13	221 ± 8
ESR (mm/h) [a]	8 ± 2	12 ± 1
creatinine (µmol/L)	68 ± 3	73 ± 2
AST (U/L)	22 ± 1	26 ± 1
ALT (U/L)	16 ± 1	23 ± 1

The values shown represent a mean ± standard error of the mean unless otherwise indicated. ESR = erythrocyte sedimentation ration, [a] performed in 37/39 healthy old subjects, AST = aspartate transaminase, ALT = alanine transaminase.

Supplemental Table 3. Survival outcome and prescribed systemic treatment after inclusion.

	young melanoma patients *n* = 13	old melanoma patients *n* = 18
median overall survival (months)	15.8 (95% CI 9.6–21.9)	16.6 (95% CI 5.7–27.6)
1-year overall survival (%)	62	59
2-year overall survival (%)	42	24
dacarbazine (*n*)	7	7
vemurafenib or dabrafenib monotherapy (*n*)	6	11
dabrafenib + trametinib (*n*)	4	5
ipilimumab (*n*)	7	2
nivolumab or pembrolizumab (*n*)	7	3
other treatment (*n*)	2	1

CI = confidence interval.
